# NSCLC molecular testing in Central and Eastern European countries

**DOI:** 10.1186/s12885-018-4023-4

**Published:** 2018-03-09

**Authors:** Ales Ryska, Peter Berzinec, Luka Brcic, Tanja Cufer, Rafal Dziadziuszko, Maya Gottfried, Ilona Kovalszky, Włodzimierz Olszewski, Buge Oz, Lukas Plank, Jozsef Timar

**Affiliations:** 1 The Fingerland Department of Pathology, Charles University Faculty of Medicine and University Hospital, Hradec Králové, Czech Republic; 2 Department of Oncology, Specialised Hospital of St Zoerardus Zobor, Nitra, Slovakia; 3 Institute of Pathology, Medical University of Graz, Graz, Austria; 4 Institute of Pathology, University of Zagreb School of Medicine, Zagreb, Croatia; 5 Medical Faculty Ljubljana, University Clinic Golnik, Golnik, Slovenia; 6 Medical University of Gdansk, Gdansk, Poland; 7 Meir Medical Center, Kfar Saba, Israel; 8 1st Institute of Pathology and Experimental Cancer Research, Semmelweis University, Budapest, Hungary; 9 Institute of Oncology, Warsaw, Poland; 10 Cerrahpasa Medical Faculty, Istanbul, Turkey; 11 Department of Pathology, Comenius University, Jessenius Medical Faculty and University Hospital, Martin, Slovakia

**Keywords:** Non-small cell lung cancer, *EGFR* mutations, *ALK* rearrangements, Molecular testing, Central eastern European region

## Abstract

**Background:**

The introduction of targeted treatments for subsets of non-small cell lung cancer (NSCLC) has highlighted the importance of accurate molecular diagnosis to determine if an actionable genetic alteration is present. Few data are available for Central and Eastern Europe (CEE) on mutation rates, testing rates, and compliance with testing guidelines.

**Methods:**

A questionnaire about molecular testing and NSCLC management was distributed to relevant specialists in nine CEE countries, and pathologists were asked to provide the results of *EGFR* and *ALK* testing over a 1-year period.

**Results:**

A very high proportion of lung cancer cases are confirmed histologically/cytologically (75–100%), and molecular testing of NSCLC samples has been established in all evaluated CEE countries in 2014. Most countries follow national or international guidelines on which patients to test for *EGFR* mutations and *ALK* rearrangements. In most centers at that time, testing was undertaken on request of the clinician rather than on the preferred reflex basis. Immunohistochemistry, followed by fluorescent in situ hybridization confirmation of positive cases, has been widely adopted for *ALK* testing in the region. Limited reimbursement is a significant barrier to molecular testing in the region and a disincentive to reflex testing. Multidisciplinary tumor boards are established in most of the countries and centers, with 75–100% of cases being discussed at a multidisciplinary tumor board at specialized centers.

**Conclusions:**

Molecular testing is established throughout the CEE region, but improved and unbiased reimbursement remains a major challenge for the future. Increasing the number of patients reviewed by multidisciplinary boards outside of major centers and access to targeted therapy based on the result of molecular testing are other major challenges.

**Electronic supplementary material:**

The online version of this article (10.1186/s12885-018-4023-4) contains supplementary material, which is available to authorized users.

## Background

Globally, for several decades, lung cancer has been the most common cancer and the leading cause of cancer deaths. The situation is particularly serious in Central and Eastern European (CEE) countries, which have the highest age-standardized incidence rates in men around the world [1]. Incidence rates in women are generally lower than in men, but are increasing in many countries worldwide. There are some geographical differences in incidence, reflecting in part the different historical exposure to tobacco smoking [1]. The diagnosis is often not made until late in the course of the disease and, as a result, only a minority of patients are cured and the ratio of mortality-to-incidence is very high. Almost 70% of patients have locally advanced or metastatic disease at initial diagnosis [2].

Nowadays, only about 15% of lung cancer cases are small cell lung cancer, with the majority of lung cancer cases classified as non-small cell lung cancer (NSCLC). When the diagnosis is made based on a small biopsy or cytology sample, besides the three common types of NSCLC (squamous cell carcinoma, adenocarcinoma, and non-small cell carcinoma not otherwise specified [NOS]) several additional subtypes can be defined by morphology, immunohistochemistry (IHC), and molecular pathology [2, 3]. Although most lung cancers are attributable to tobacco smoking, approximately 10–15% of cases in Western countries occur in lifelong never-smokers and these are almost exclusively adenocarcinomas [4].

The study of molecular biology of NSCLC has had a major impact on diagnosis and treatment of this disease [5–7]. The work of the Lung Cancer Mutation Consortium and other groups has shown that driver mutations or other oncogene alterations are present in more than half of all adenocarcinomas [8]. The discovery of targetable genetic alterations, such as activating mutations of the epidermal growth factor receptor (*EGFR*) and anaplastic lymphoma kinase (*ALK*) rearrangements, has led to the implementation of precision therapy for certain subtypes of lung cancer based on appropriate patient selection [9]. Clinical trials have shown significantly longer progression-free survival in patients with *EGFR* mutations who are treated with EGFR tyrosine kinase inhibitors (TKIs) compared with chemotherapy [10]. Similarly, ALK TKI treatment of patients with *ALK*-rearranged tumors prolongs progression-free survival compared with first-line chemotherapy [11]. Patients with a druggable molecular alteration who are treated with a corresponding targeted treatment benefit from significantly higher response rates and longer progression-free survival, although an improvement in overall survival with targeted agents has not been shown by the majority of randomized clinical trials [8]. There are probably multiple reasons for the lack of overall survival advantage seen in clinical trials, one of the most important reasons is a high number of patients crossing over to targeted agents after failure of treatment in the chemotherapy arms.

This move towards biomarker-based treatment approaches has highlighted the importance of accurate molecular diagnosis. In addition to classical morphologic classification, molecular analysis of tumor samples is essential to determine if a druggable oncogenic alteration is present. Consequently, the pathologist is now a key member of the multidisciplinary lung cancer team [12].

Identification of the challenges for personalized lung cancer treatment within the CEE region might facilitate molecular diagnostics and improve patient care. Information about molecular testing practices for NSCLC in the CEE region is relatively limited. The INSIGHT study has provided some information on *EGFR* mutation rates, testing, and compliance with testing guidelines in several Central European countries [13]. However, only very few data from this region are available on *ALK* testing. Our study was designed to collect information on both *EGFR* and *ALK* testing from a large number of CEE countries.

## Methods

A Working Group of oncologists, pulmonologists, and pathologists from the CEE region was established to obtain more information on NSCLC molecular testing in their countries and to raise awareness of the current issues around personalized medicine for lung cancer.

As a first step, a questionnaire (Additional file 1) with 37 questions addressing issues of molecular testing and NSCLC management was distributed in the second quarter of 2014 to 59 specialists (epidemiologists, oncologists, pulmonologists, and pathologists) from nine CEE countries. In June 2015, pathologists were also asked to provide details of the results of *EGFR* and *ALK* testing over a 1-year period.

## Results

### Respondents

There were a total of the 42 responses from nine countries; the number of responders by country are shown in Table 1 (data were not available for some questions; see Additional file 1 for the questionnaire).

**Table 1:** Countries participating and number of responders

Bulgaria	2
Croatia	4
Czech Republic	4
Hungary	8
Israel	3
Poland	7
Slovakia	7
Slovenia	3
Turkey	4

### Lung cancer types

Table 2 shows the proportion of lung cancer cases that are confirmed morphologically in each country.

**Table 2:** Lung cancer cases morphologically confirmed

Country (number of responses)	Proportion of cases, %
Bulgaria	75*
Croatia	100*
Czech Republic	85^†^
Israel	100*
Slovakia	83*
Slovenia	92*

*Registry data; ^†^Best estimate

**Fig. 1:**
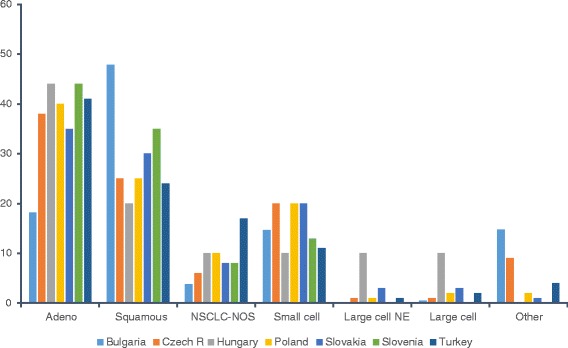
Lung cancer subtypes by country. NE, neuroendocrine carcinoma; NOS, not otherwise specified; NSCLC, non-small cell lung cancer

Figure 1 shows the breakdown of lung cancer types for selected countries. Adenocarcinoma is the most common type in all countries except Bulgaria, whereas small cell lung cancer represented between 10% and 20% of cases in all countries.

### *EGFR* testing

Most countries have national guidelines or follow European guidelines on *EGFR* mutation testing [2]. European guidelines are followed in Bulgaria, Croatia, Israel, Slovakia, Slovenia, and Turkey. Bulgaria, Czech Republic, Hungary, Israel, Poland, and Slovakia also have national guidelines, whereas some centers in Poland follow local guidelines. Some Turkish centers follow American College of Pathologists guidelines [12].

Most centers reported that adenocarcinomas and NSCLC-NOS were tested for activating *EGFR* mutations. In some countries (Bulgaria, Czech Republic, Israel, Slovakia, Slovenia, and Turkey), large cell and squamous cell tumors may be tested in selected cases (mostly on request from the treating physician). In most countries, only advanced tumors (stage IIIb and IV) were generally tested for *EGFR* mutations. In the Czech Republic, Slovakia, and Slovenia, as well as in certain individual centers in other countries, other stages were also tested for research purposes.

In most centers, *EGFR* mutation testing was undertaken when requested by the clinician, usually the oncologist treating the patient. In the Czech Republic, Slovakia, and Slovenia, and some centers in Croatia, testing was reflex (i.e. the pathologist automatically tested all tumors that met the criteria). In Hungary, it is policy to test for *KRAS* mutations first, and the presence of a *KRAS* mutation is an exclusion criterion for *EGFR/ALK* testing. In Turkey, *EGFR/ALK* testing is not performed in *KRAS* mutation-positive tumors, although *KRAS* testing is not routine.

In many countries (Croatia, Czech Republic, Hungary, Israel, Slovakia, and Slovenia), at least 65% of eligible tumors were actually tested for *EGFR* mutations in 2014. However, a significant proportion of samples (5–25%) were inadequate for testing, usually because the sample was too small or did not contain enough tumor cells.

Usually, more than one method was used in each country for *EGFR* mutation testing. Real-time polymerase chain reaction (PCR) was used in all countries, direct sequencing in five countries, and other methods were used in addition in only two countries.

The incidence of specific *EGFR* mutations in selected centers in 2014 is shown in Table 3; the frequency of *EGFR*-mutated tumors ranged from 6.7 to 15.2%. These numbers cannot be compared because of the different inclusion criteria for testing.

**Table 3:** Results of *EGFR* testing in selected centers in 2014^a^

	*EGFR,* n (%)					Non-diagnostic
	WT	Mut exon 18	Mut exon 19	Mut exon 20	Mut exon 21	
Croatia (Zagreb)	561 (85.9)	8 (1.2)	46 (7)	8 (1.2)	30 (4.6)	11
Czech (Prague)	154 (90.6)	4 (2.4)	6 (3.5)	2 (1.2)	4 (2.2)	15
Czech (Hradec Kralove)	234 (90.0)	0	15 (5.8)	2 (0.8)	9 (3.5)	6
Hungary (Budapest, Timár)	350 (85.6)	4 (1.0)	22 (5.4)	14 (3.9)	19 (4.6)	57
Hungary (Budapest, Toth)	251 (93.3)	0	9 (3.3)	1 (0.4)	8 (3.1)	2
Hungary (Budapest, Kovalszky)	500 (88.5)	6 (1.1)	27 (4.8)	0	32 (5.7)	–
Hungary (Debrecen)	760 (89.6)	0^†^	61 (7.2)	0^†^	27 (3.2)	13
Hungary (Pécs)	112 (86.8)	0	10 (7.8)	0	7 (5.4)	–
Hungary (Szeged)	617 (92.6)	4 (0.6)	27 (4.1)	2 (0.3)	16 (2.4)	71
Slovakia	361 (87.6)	1 (0.2)	30 (7)	2 (0.5)	16 (3.8)	–
Slovenia	464 (86)	5 (0.9)	39 (7.2)	8 (1.5)	25 (4.6)	57
Turkey (Cerrahpaşa)	714 (87.9)	17 (2.1)	52 (6.4)	3 (0.4)	26 (3.2)	38

*EGFR* epidermal growth factor receptor, *WT* wild-type

^a^Note that these numbers cannot be compared directly because of the different criteria for selection of samples to test

^†^Exons 18 and 20 not tested

### *ALK* testing

*ALK* testing is available in all countries except Bulgaria. Most countries have national guidelines or follow European guidelines on which subtypes to test for *ALK* rearrangements. European guidelines [2] are followed in Croatia, Slovenia, and Turkey, whereas Czech Republic, Hungary, Israel, Poland, and Slovakia follow national guidelines. In all centers, adenocarcinomas and NSCLC-NOS were tested, usually on request from the treating clinician. As for *EGFR* testing, reflex *ALK* testing for all eligible patients was implemented in the Czech Republic, Slovakia, and Slovenia.

In most countries, as for *EGFR* testing, only advanced tumors (stage IIIb and IV) were tested for *ALK* rearrangements. At some centers in Croatia, Czech Republic, Slovakia, and Slovenia, other stages were also tested for research purposes. The presence of a known *EGFR* mutation was an exclusion criterion for *ALK* testing in all countries. In addition, in countries where some or all samples are tested for *KRAS* mutations (Turkey, Slovenia, Hungary, and Poland), the presence of a *KRAS* mutation is also an exclusion criterion. For *ALK* testing, IHC followed by fluorescent in situ hybridization (FISH) and/or FISH alone were used in all countries. In Israel, other methods, including next-generation DNA sequencing, were also used (Table 4).

**Table 4:** *ALK* testing methods

Croatia (Zagreb)	IHC (IHC followed by FISH)
Czech (Hradec Kralove)	IHC, IHC followed by FISH
Czech (Prague)	IHC, IHC followed by FISH, FISH
Hungary (Budapest, Timár)	FISH
Hungary (Budapest, Toth)	FISH
Hungary (Budapest, Kovalszky)	FISH, other
Hungary (Debrecen)	FISH
Hungary (Pécs)	IHC followed by FISH
Hungary (Szeged)	FISH
Israel	IHC, IHC followed by FISH, FISH, sequencing
Poland (Warsaw)	FISH
Slovakia	IHC followed by FISH; FISH
Slovenia	IHC followed by FISH
Turkey (Cerrahpaşa)	IHC followed by FISH

*FISH* fluorescent in situ hybridization, *IHC* immunohistochemistry

The incidence of *ALK* rearrangements determined in selected centers in 2014 is shown in Table 5. The substantial differences in the frequency, which ranged from 1.6% to 12%, are most probably due to different testing approaches, excluding *KRAS-* and *EGFR*-positive cases from *ALK* testing or not.

**Table 5:** Results of *ALK* testing in selected centers in 2014^a^

	*ALK,* n (%)		Non-diagnostic
	WT	Rearrangement	
Croatia	161 (93.1)	12 (6.9)	28
Czech (Prague)	55 (96.5)	2 (3.5)	10
Czech (Hradec Kralove)	260 (95.2)	13 (4.8)	2
Hungary (Budapest, Timár)	332 (94.9)	18 (5.1)	0
Hungary (Budapest, Toth)	122 (98.4)	2 (1.6)	4
Hungary (Budapest, Kovalszky)	415 (95.6)	19 (4.4)	0
Hungary (Debrecen)	226 (91.5)	21 (8.5)	35
Hungary (Pécs)	55 (94.8)	3 (5.2)	2
Slovakia	375 (88)	51 (12)	0
Slovenia	199 (96.5)	7 (3.5)	0
Turkey (Cerrahpaşa)	764 (95.6)	35 (4.4)	14

*ALK* anaplastic lymphoma kinase, *WT* wild-type

^a^Note that these numbers cannot be compared directly because of the different criteria for selection of samples to test; in some centers only samples negative for *KRAS* and *EGFR* mutations were tested for *ALK* translocations

### Testing for other mutations

Tumor samples were tested routinely for *KRAS* mutations in Hungary and Slovenia; some samples were tested in Turkey and Poland. *ROS1* testing was routine in Slovenia and was undertaken on request in Slovakia.

### Reimbursement for molecular testing

There are several different sources of funding for *EGFR* and *ALK* testing in the region. In the Czech Republic, Hungary, Israel, Poland, Slovakia, Slovenia, and Turkey, testing is partly or fully reimbursed by the national health authority/national health insurance. Private insurance covers some testing in Israel and Turkey.

The pharmaceutical industry supported some testing in Hungary, Poland, and Slovenia, and was the only source of financial support for testing in Bulgaria (*EGFR* only) and Croatia (*EGFR* and *ALK*). The industry did not finance testing in Czech Republic, Israel, Slovakia, or Turkey. However, in the personal experience of the authors, although the molecular testing is stated to be fully reimbursed, this is not the case in practice. Often, there are various forms of budget capping with a limitation on the number of tests performed (e.g. based on the number of samples tested in the previous period). This policy is a disincentive to reflex testing. In Hungary, for example, only 30% of tests were reimbursed.

### Multidisciplinary approach

Multidisciplinary lung cancer teams/tumor boards are established in all countries; however, these are often only functioning fully as part of routine clinical practice at specialized lung cancer treatment centers. In Hungary, Poland, and Slovenia, it is mandatory for all cases to be discussed by a multidisciplinary tumor board. In Turkey, it is obligatory for selected cases. When multidisciplinary teams/tumor boards are operational, a pathologist is usually a member.

The proportion of NSCLC cases actually discussed at a multidisciplinary tumor board is 75–100% at most specialist centers; however, there is wide variation and can be as low as 20% in some hospitals. There was a trend towards a higher proportion of cases being discussed at multidisciplinary tumor boards at respondents’ own centers compared with their estimates for the country as a whole. Data on how many patients with druggable EGFR or ALK alterations get access to targeted drugs were not collected, however according to the personal experience of the authors, access to EGFR and/or ALK TKIs is often limited, mainly due to local reimbursement policy restrictions.

## Discussion

The survey confirmed that a very high proportion of lung cancer cases are verified by histology or cytology (75–100%) in CEE countries and that, in most countries, the data can be derived from the established cancer registries. Adenocarcinoma is the most common type in all countries except Bulgaria, whereas 10–20% of cases were small cell lung cancer in all countries, which is consistent with global data [14]. The high incidence of squamous cell carcinoma in Bulgaria may reflect the high levels of smoking, relatively late introduction of filtered cigarettes, and pollution levels [15].

It is encouraging that molecular testing of NSCLC samples has been established in all CEE countries evaluated in 2014, and that most countries follow national or international guidelines on which patients to test for *EGFR* mutations and *ALK* rearrangements. However, in most centers, *EGFR* and *ALK* testing was undertaken on request of the clinician, rather than automatically for eligible samples (reflex testing), an approach which can lead to delays in availability of test results and in the initiation of targeted treatment. There is an increasing focus on shortening the turnaround time for test results, and incorporation of reflex testing at the level of the pathologist can help to avoid such delays [16].

The results showed that a significant proportion of samples were unsuitable for testing for various reasons. Various initiatives, including better communications as well as educational initiatives directed at physicians who collect tissue samples, may improve the situation and help to ensure that samples are of sufficient size and quality for molecular testing [16].

The results of molecular testing presented here (Tables 3 and 5) provide interesting insights into the frequency of *EGFR* mutations and *ALK* rearrangements in the region. Yet only very limited general conclusions can be drawn on the overall frequency of *EGFR* mutations and *ALK* translocations because of the differences between centers in testing policy, selection of samples for testing based on clinical factors (i.e. not all samples were tested) and sequential testing at some centers resulting in *ALK* testing being performed only on EGFR- and KRAS-negative samples. Consistent with published data, most *EGFR* mutations reported were in exons 19 and 21 [17, 18]. Information was not requested on *KRAS* mutation rate, which would have been particular relevant for Hungary where testing was routine. However, a recent paper reported that the incidence of *KRAS* mutations was 28.6% in 532 consecutive Caucasian patients tested at Semmelweis University [19].

The results show that IHC, followed by FISH confirmation in positive cases, had been widely adopted for *ALK* testing in the region in 2014. FISH testing was still regarded as the gold standard for *ALK* testing; however, this method is relatively costly, time-consuming, and technically difficult to perform for routine use, which has led to extensive evaluation of IHC as an alternative used for screening purposes [20–22]. Data published after our survey showed that both D5F3 and 5A4 antibodies are able to detect *ALK* rearrangements reliably and are equally well suited for routine diagnostic use [23]. Several studies have shown good concordance between the results of IHC and FISH for *ALK* testing [24–27]. Indeed, some investigators have shown that IHC can be useful in cases with atypical or borderline FISH results [26, 28].

Acceptance by the United States Food and Drug Administration (FDA) of the Ventana ALK D5F3 IHC as a companion test to identify patients for crizotinib treatment provides additional support for the routine use of IHC. The test provides a fast and accurate method to identify ALK protein expression, with a binary scoring system, and has been validated clinically by retrospective testing of tissue samples from patients screened for inclusion in crizotinib clinical trials [29]. These promising data encourage some centers to use IHC as a primary method replacing FISH. Immunohistochemistry can readily be applied to tissue samples, cell blocks prepared from effusions and Papanicolaou-stained cytologic slides [22, 27].

The European consensus recommended that all non-squamous NSCLC tumors in patients with advanced/recurrent disease be tested for *EGFR* mutations and *ALK* translocations. Selected squamous tumors (from patients with minimal or remote smoking history) should be strongly considered for testing. Sequential testing is not recommended and parallel testing of multiple mutations on the same sample is becoming the standard [2]. More recent European recommendations are consistent with these statements. Sanger sequencing, pyrosequencing, and next-generation sequencing are recommended for *EGFR* testing and validated tests including FISH and IHC may be used for ALK testing [30]. Real-time PCR is also widely used. National guidelines, where they exist, are broadly consistent with these recommendations; in Hungary, *KRAS* testing is performed before testing for other genetic alterations.

With the increasing number of molecular markers that need to be examined for optimal selection of targeted treatment, the cost of up-to-date molecular testing is rapidly growing. The financial burden of testing the entire cohort of eligible patients can, in fact, reach the cost of the treatment of several individual patients identified by the testing, namely in tumors driven by rare mutations [31]. Thus, routine use of modern approaches, such as next-generation sequencing resulting in dramatic reduction of testing costs, is eagerly awaited.

It should be noted that the data discussed here reflect the status quo in 2014, and molecular testing is evolving fast with changes in testing methods being implemented. The growing use of IHC for *ALK* testing has already been discussed. In addition, many laboratories in Europe are now adopting next-generation sequencing that can be applied to formalin-fixed paraffin-embedded tissue in routine diagnostic practice. This allows for the detection of many genetic alterations and oncogene targets in parallel, providing the opportunity for fast and deep characterization of tumors as well as for the potential for other targeted therapies [32].

Adherence to the best practices in molecular testing is crucial to ensure accurate diagnoses and appropriate clinical decisions [30, 33]. Quality control is essential to ensure consistent and reliable molecular diagnostic results and to facilitate comparison of results from different laboratories. External quality assessment (EQA) programs have been established for both *EGFR* and *ALK* testing [33, 34]. The vast majority of the laboratories contributing to this study already participate in EQA programs. Many laboratories in the region participate in the European Society of Pathology Lung External Quality Assessment Scheme [19]. As the results of molecular testing directly influence the management of individual patients, EQA is essential to guarantee optimal quality of testing. Therefore, each laboratory should prove successful participation in the appropriate EQA program to be included to the network of testing centers [35].

Multidisciplinary tumor boards play a key role in optimizing the diagnosis and treatment of lung cancer [30]. With rapid progress in the molecular profiling of NSCLC and its increasing complexity, it is essential that the tumor boards include specially trained molecular pathologists, as well as molecular biologists, among the tumor board members when discussing the best possible treatment for each individual patient with NSCLC. Although it is positive that all countries studied have implemented lung cancer tumor boards, these are more likely to be operational only in specialized lung cancer treatment centers.

## Conclusions

Non-small cell lung cancer molecular testing is established in all CEE countries participating in this study. The responses show that all countries follow guidelines regarding *EGFR* and *ALK* testing, with most countries testing only advanced stages of adenocarcinomas, NSCLC-NOS, and NSCLC when an adenocarcinoma component cannot be excluded. Most countries are still undertaking testing on request and not implementing the preferred reflex policy.

Limited reimbursement is a significant barrier to molecular testing in the region and a disincentive to reflex testing. The authors recommend that testing should be independently funded.

The results show that ensuring adequate NSCLC samples and enabling wide access of eligible patients to molecular testing are key issues for the future. Increasing the number of patients reviewed by multidisciplinary boards and the access of patients with druggable molecular alterations to targeted drugs are other major challenges.

## Additional file

Additional file 1: Questionnaire addressing issues of molecular testing and NSCLC management. Questionnaire was provided to 59 specialists (epidemiologists, oncologists, pulmonologists, and pathologists) from nine CEE countries requesting information. (PDF 191 kb)

## Abbreviations

ALK: Anaplastic lymphoma kinase
CEE: Central and Eastern European
EGFR: Epidermal growth factor receptor
EQA: External quality assessment
FDA: Food and Drug Administration
FISH: Fluorescent in situ hybridization
IHC: Immunohistochemistry
NOS: Not otherwise specified
NSCLC: Non-small cell lung cancer
PCR: Polymerase chain reaction
TKI: Tyrosine kinase inhibitor
