# Supplementary material for: NSCLC molecular testing in Central and Eastern European countries
Source: BMC Cancer. 2018 Mar 9;18:269. doi: 10.1186/s12885-018-4023-4 (PMC5845184; doi:10.1186/s12885-018-4023-4)
Supplement: Supplementary file 1 — Questionnaire addressing issues of molecular testing and NSCLC management. Questionnaire was provided to 59 specialists (epidemiologists, oncologists, pulmonologists, and pathologists) from nine CEE countries requesting information. (PDF 191 kb) [file 12885_2018_4023_MOESM1_ESM.pdf]

Regional NSCLC Working Group: Position paper questionnaire for national experts

Thank you for your interest in completing this survey. Please take a few minutes to read the following instructions.

How to complete the survey
To ensure that the information included within the manuscript is as accurate as possible, we ask that you base your answers on actual data (i.e. information extracted from patient records at your own hospital, national registry data or published cancer statistics). If you cannot provide specific data, we kindly ask that you provide your **best estimate** based on your expert knowledge. You may also be asked to indicate the **source(s)** of the data you provide.

How to complete the questionnaire

- To complete the survey, please use either Adobe Acrobat, or the free Adobe Acrobat Reader (available to download for Windows, or Macintosh from https://get.adobe.com/uk/reader/otherversions/ or from iTunes for iOS and the Google Play store for Android)
  - If you are using a 3rd party program (non-Adobe), then please see option (b) below for instructions on how to submit the completed questionnaire
- Where indicated, please provide the either letter (a, b, c etc.) or numerical value in the boxes indicated; also, where indicated, please provide your free text answers

How to return the information to us

(a) Using Adobe Acrobat/ Adobe Acrobat reader:

- Once you have completed the questionnaire please click on the 'Submit form' option that is located the bottom of the survey
- You will be asked if you wish to connect to our secure server (via adobe.com); please click 'OK' and your response will automatically be delivered to the survey inbox

(b) Using non-Adobe software

- Please complete the questions as normal
- To submit the questionnaire, please save the form as a pdf file, and email to NSCLCPositionPaper@choicehs.com
- If saving a pdf is not possible, then please complete the survey, print it, scan the printout and return the scanned images to NSCLCPositionPaper@choicehs.com

Demographics:
(Boxes in red are mandatory)

Respondent name:

Institution/Department:

Town/City and Country:

Specialty/job description:

NSCLC Epidemiology

Q1: Does **your country** have a population-based cancer registry recording data on NSCLC incidence of NSCLC?

a. Yes, national registry<br>b. Yes, regional registry<br>c. No<br>d. Don't know	**Please enter your choice:**<br>If **yes**, please provide registry name(s):

Q2: What proportion of NSCLC patients **in your country** have Stage IIIB/IV disease at diagnosis?

**Answer based on data records**	**Best estimate**
Please indicate a percentage: % <br>What is the source of these data?<br>a. Cancer registry (provide details)<br>b. Other source(s)<br>**Source:**<br>If **other source(s)**, please provide a reference for these data:	Please indicate a percentage: %

Diagnosis of NSCLC

Q3: In the last year for which data are available, how many cases of NSCLC (any stage) were diagnosed **in your country**?

**Answer based on data records**	**Best estimate**
Year:<br><br>Please indicate the number:<br><br>What is the source of these data?<br>a. Cancer registry (provide details)<br>b. Other source(s)<br>**Source:**<br>If **other source(s)**, please provide a reference for these data:	Year:<br><br>Please indicate an approximate number:

Q4: In 2012, how many cases of NSCLC (any stage) were seen **at your centre**?

**Answer based on data records**	**Best estimate**
Please indicate the number:<br><br>What is the source of these data?<br>a. Hospital database/patient records<br>b. Other source(s)<br>**Source:**<br>If **other source(s)**, please provide a reference for these data:	Please indicate an approximate number:

Q5: Of these cases, what proportion was diagnosed using tissue material originating from the following sources (answer should total 100%):

Answer based on data records	Best estimate
a. **Cytology sample only:** %<br>(Sputum cytology, brush cytology, FNA or pleural fluid aspiration)<br>b. **Tissue sample only:** %<br>(Transbronchial biopsy, bronchial, pleural or core biopsies, mediastinoscopy, lymph node excision, VATS biopsy/resection, thoracotomy tumour excision)<br>c. **Concurrent cytology sample + solid tissue sample:** %<br>d. **No microscopic diagnosis:** %<br><br>What is the source of these data?<br>a. Hospital database/patient records<br>b. Other source(s)<br>**Source:**<br>If **other source(s)**, please provide a reference for these data:	a. **Cytology sample only:** %<br>(Sputum cytology, brush cytology, FNA or pleural fluid aspiration)<br>b. **Tissue sample only:** %<br>(Transbronchial biopsy, bronchial, pleural or core biopsies, mediastinoscopy, lymph node excision, VATS biopsy/resection, thoracotomy tumour excision)<br>c. **Concurrent cytology sample + solid tissue sample:** %<br>d. **No microscopic diagnosis:** %

Q6: For each stage of disease shown below, please indicate the proportion of NSCLC patients **at your centre** for whom a surgical sample from the primary tumour is available?

Answer based on data record	Best estimate
a. Stage I: %<br>b. Stage II: %<br>c. Stage IIIA: %<br>d. Stage IIIB/IV: %<br><br>What is the source of these data?<br>a. Cancer registry<br>b. Other source(s)<br>**Source:**<br>If **other source(s)**, please provide a reference for these data:	a. Stage I: %<br>b. Stage II: %<br>c. Stage IIIA: %<br>d. Stage IIIB/IV: %

Q7: How many hospitals **in your country, where lung cancer is treated,** have an in-house pathology department that routinely performs morphological diagnostics (cytology and/or histopathology) of lung cancer?

Answer based on data records	Best estimate
**Please indicate:**<br>No. of hospitals treating lung cancer with a pathology department:<br><br>Of these, total no. of pathology labs performing cyto/histological diagnosis of NSCLC:<br>Total no. of hospitals in your country:<br><br>What is the source of these data?<br>a. National health statistics<br>b. Other source(s)<br>**Source:**<br>If **other source(s)**, please provide a reference for these data:	**Please indicate:**<br>No. of hospitals treating lung cancer with a pathology department:<br><br>Of these, total no. of pathology labs performing cyto/histological diagnosis of NSCLC:<br>Total no. of hospitals in your country:

Q8: In what proportion of NSCLC cases tested by your laboratory in 2012 was immunohistochemistry used to confirm/finalise the histological diagnosis (TTF1, p63, etc.)?

Answer based on data records	Best estimate
Please indicate a percentage: %<br><br>What is the source of these data?<br>a. Hospital database/patient records<br>b. Other source(s)<br>**Source:**<br>If **other source(s)**, please provide a reference for these data:	Please indicate a percentage: %

Q9: What proportion of the lung cancer cases diagnosed **in your country** are classified as the following histological subtypes?

Answer based on data records	Best estimate
a. Adenocarcinoma: %<br>(incl. NSCLC, favour adenocarcinoma)<br>b. Squamous cell carcinoma: %<br>(incl. NSCLC, favour squamous cell carcinoma)<br>c. NSCLC-NOS: %<br>d. Small cell carcinoma: %<br>e. Large cell neuroendocrine: %<br>f. Large cell carcinoma: %<br>g. Other: %<br><br>What is the source of these data?<br>a. Cancer registry<br>b. Other source(s)<br>**Source:**<br>If **other source(s)**, please provide a reference for these data:	a. Adenocarcinoma: %<br>(incl. NSCLC, favour adenocarcinoma)<br>b. Squamous cell carcinoma: %<br>(incl. NSCLC, favour squamous cell carcinoma)<br>c. NSCLC-NOS: %<br>d. Small cell carcinoma: %<br>e. Large cell neuroendocrine: %<br>f. Large cell carcinoma: %<br>g. Other: %

Molecular testing practices for NSCLC

EGFR testing

Q10: Are NSCLC patients **in your country** currently tested for *EGFR* mutation status?

a. Yes, all of them<br>b. Yes, some of them<br>c. No<br>d. Don't know	**Choice:**<br>If your answer is "**No**", please give the reason:

Q11: How is EGFR testing **in your country** currently funded? (Please specify all that apply)

a. National healthcare authority<br>b. National health insurance company<br>c. Private insurance<br>d. Pharmaceutical industry<br>e. Other source(s); please provide details:	**Please specify all that apply:**

Q12: Which histological subtypes of NSCLC are tested for *EGFR* mutation status **in your country**? (Please specify all that apply)

Answer based on data records	Best estimate
a. Adenocarcinoma (incl. NSCLC, favour adenocarcinoma)<br>b. NSCLC-NOS<br>c. Squamous cell carcinoma (incl. NSCLC, favour squamous cell carcinoma)<br>d. Large cell neuroendocrine carcinoma<br>e. Other (please specify)<br>**Choice:**<br>If other (e), please specify:	a. Adenocarcinoma (incl. NSCLC, favour adenocarcinoma)<br>b. NSCLC-NOS<br>c. Squamous cell carcinoma (incl. NSCLC favour squamous cell carcinoma)<br>d. Large cell neuroendocrine carcinoma<br>e. Other (please specify)<br>**Choice:**<br>If other (e), please specify:

Q13: Does **your country** follow guidelines to select which histological subtypes of NSCLC undergo EGFR testing?

a. Yes, national testing guidelines<br>b. Yes, European testing guidelines<br>c. Yes, local guidelines<br>d. Yes, other guidelines<br>e. No, we have no guidelines<br>f. No, we cannot follow them<br>(due to financial limitations, tissue availability, lack of cooperation, etc.)<br>**Choice:**<br>If you follow guidelines to guide EGFR testing, please provide a reference:

Q14: Does **your centre** follow guidelines to select which histological subtypes of NSCLC undergo EGFR testing?

a. Yes, national testing guidelines<br>b. Yes, European testing guidelines **Choice:**<br>c. Yes, local guidelines<br>d. Yes, other guidelines<br>e. No, we have no guidelines<br>f. No, we cannot follow them<br>(due to financial limits, tissue availability, lack of cooperation, etc.)<br><br>If you follow guidelines to guide EGFR testing, please provide a reference:

Q15: Which stages of NSCLC are tested for *EGFR* mutation status at initial diagnosis **at your centre**?

**Answer based on data records**	**Best estimate**
a. All stages<br>b. Stage IIIB and stage IV only<br>c. Do not know<br>**Choice:**<br>What is the source of these data?<br>a. Laboratory records/patient records<br>b. Other source(s)<br>**Source:**<br>If **other source(s)**, please provide a reference for these data:	a. All stages<br>b. Stage IIIB and stage IV only<br>c. Do not know<br>**Choice:**

Q16: Which strategy is used for EGFR testing?

a. Reflex testing: every patient is tested automatically (based on histological type)<br>b. On-demand: requested by the clinician who took the biopsy<br>c. On-demand: requested by clinician who is treating the patient (e.g. oncologist)<br>d. Other strategy (please specify):<br>**Choice:**

Q17: In what proportion of the NSCLC cases which are eligible for EGFR testing **in your country** is testing actually performed?

**Answer based on data records**	**Best estimate**
Indicate a percentage: %<br><br>What is the source of these data?<br>a. National cancer statistics<br>b. Other source(s)<br>**Source:**<br>If **other source(s)**, please provide a reference for these data:	Indicate a percentage: %

Q18: In what proportion of the NSCLC cases eligible for testing **at your centre** is EGFR testing actually performed?

**Answer based on data records**	**Best estimate**
Indicate a percentage: % <br>What is the source of these data? a. Hospital database/patient records b. Other source(s) **Source:** If **other source(s)**, please provide a reference for these data:	Indicate a percentage: %

Q19: Please list any exclusion criteria inclusion/exclusion criteria for EGFR testing in your country? E.g. gender, smoking history, performance status, KRAS status, etc.

Q20: Where is EGFR testing for the NSCLC patients treated **at your centre** performed?

a. In-house testing laboratory   b. External national laboratory   c. External laboratory (outside of your country)   d. External national commercial laboratory   e. External international commercial laboratory (outside of your country)   **Choice:**   If external, please provide details: (name/location of the testing laboratory)

Q21: If EGFR testing is performed in-house, what test method(s) do you use?

a. Direct sequencing b. Immunohistochemistry with mutation-specific antibody c. Real-time PCR (e.g. ARMS, TheraScreen®, Cobas®) d. Pyrosequencing e. Other method f. I do not know what method is used	**Please specify all that apply:** <br><br><br>**If other (e), please specify:**

Q22: If EGFR testing is performed in-house, what proportion of tissue specimens are inadequate for testing?

**Answer based on data records**	**Best estimate**
Please indicate a percentage: % <br>What is the source of these data? a. Hospital database/patient records b. Other source(s) **Source:** If **other source(s)**, please provide a reference for these data:	Please indicate a percentage: %

Q23: Please indicate the reason(s) why specimens are inadequate (answer should total 100%)

Answer based on data records	Best estimate
a. Tissue specimen too small: % b. Inadequate fixation/storage of tissue specimen: % c. Not enough tumour cells in the tissue sample: % d. Other reason(s): % <br>What is the source of these data? a. Hospital database/patient records b. Other source(s) **Source:** If **other source(s)**, please provide a reference for these data:	a. Tissue specimen too small: % b. Inadequate fixation/storage of tissue specimen: % c. Not enough tumour cells in the tissue sample: % d. Other reason(s): %

Q24: What is the average turnaround time of EGFR testing **for your patients**? (I.e. time from when specimen is sent to the laboratory for analysis until final test results are received.) **Indicate average number of days:**

ALK testing

Q25: Is ALK testing **available** for NSCLC patients **in your country**?

a. Yes; b. No; c. Do not know	**Choice:**

Q26: Are NSCLC patients **in your country** currently tested for *ALK* rearrangements?

a. Yes, all of them
b. Yes, some of them selected by histology (indicate histological subtypes that are tested):
   i. Adenocarcinoma (incl. NSCLC, favour adenocarcinoma)
   ii. NSCLC-NOS
   iii. Squamous cell carcinoma (incl. NSCLC favour squamous cell carcinoma)
   iv. Large cell neuroendocrine carcinoma
   v. Other
c. No
d. Don't know

Choice: **Please specify subtypes (b; i–iv):**

If answer is "**No**", please give the reason:

Q27: How is ALK testing currently funded **in your country**? (Please specify all that apply)

a. National healthcare authority b. National health insurance company c. Private insurance d. Pharmaceutical industry e. Other source(s)	**Choices:** <br>**Details:**

Q28: Please list any other inclusion/exclusion criteria for ALK testing E.g. gender, smoking history, performance status, EGFR/KRAS status, etc.

Q29: Does **your centre** follow guidelines to select which histological types of NSCLC undergo ALK testing?

a. Yes, national testing guidelines<br>b. Yes, European testing guidelines<br>c. Yes, local guidelines<br>d. Yes, other guidelines<br>e. No, we have no guidelines<br>f. No, we cannot follow them<br>(due to financial limitations, tissue availability, lack of cooperation, etc.)	**Choice:**<br>**Please provide a reference:**

Q30: What strategy is used for ALK testing?

a. Reflex testing – every patient is tested automatically, based on histological type
b. On-demand – requested by the clinician who took the biopsy
c. On-demand – requested by clinician who is treating the patient (oncologist)
d. Other strategy

Choice:

If other (d), please specify:

Q31: Which stages of NSCLC are tested for *ALK* rearrangements at the time of the initial diagnosis **at your centre**?

**Answer based on data records**	**Best estimate**
a. All stages<br>b. Stage IIIB and stage IV only<br>c. Do not know<br>**Choice:**<br><br>What is the source of these data?<br>a. Laboratory records/patient records<br>b. Other source(s)<br>**Source:**<br>**Reference:**	a. All stages<br>b. Stage IIIB and stage IV only<br>c. Do not know<br>**Choice:**

Q32: What method(s) are currently used for ALK testing **in your country**?
(Please specify all that apply, and indicate details of specific antibodies or kits used)

	**Please indicate the details of specific antibodies or kits used by each option that applies**
a. Immunohistochemistry	
b. Immunohistochemical screening, followed by FISH	
c. FISH	
d. RT-PCR	
e. DNA sequencing	
f. Other method, please specify:	

Q33: Are lung cancer patients **in your country** tested for any other molecular biomarkers (e.g. KRAS, MET)? **Please provide details below, including the inclusion/exclusion criteria**

Multidisciplinary approach to lung cancer diagnosis and treatment

Q34: In your country, are multidisciplinary lung cancer teams established in routine clinical practice?

a. Yes
b. No
c. Yes, but only in specialised lung cancer treatment centres
d. Do not know

Choice:

Q35: In your country, is it mandatory for all lung cancer cases to be discussed by a multidisciplinary tumour board before any primary treatment is initiated?

a. Yes, according to local guidelines
b. Yes, according to local practice
c. No
d. Selected cases only
e. Do not know

Choice:

Q36: In reality, what proportion of lung cases are actually discussed at the multidisciplinary tumour board **in your country**?

Best estimate: **%**

Q37: In reality, what proportion of lung cancer cases are actually discussed at the multidisciplinary tumour board **at your centre**?

Best estimate: **%**

Thank you for taking the time to complete the survey, your answers are extremely valuable and will contribute to knowledge surrounding molecular testing in Central and Eastern Europe.

- **Please now submit the completed questionnaire using either the 'Submit Form' option below (for Adobe Acrobat/Acrobat Reader only), or save this pdf and email it to NSCLCPositionPaper@choicehs.com**

Submit
